# Correction: MiR-132 enhances proliferation and migration of HaCaT cells by targeting TIMP3

**DOI:** 10.1039/d0ra90075c

**Published:** 2020-07-08

**Authors:** Lina Jiang, Yizhou Jiang, Xiaohui Ji, Jiangtao Li, Xiaomei Zhai

**Affiliations:** Department of Plastic Surgery, The First Affiliated Hospital of Zhengzhou University No. 1, East Jianshe Rd Zhengzhou 450052 China qinhzhouygwcshan@163.com +86-371-66913114; Department of Breast Surgery, Shanghai Cancer Center, Fudan University Shanghai 200032 China; Department of Pathology China; Department of Breast Surgery, The People’s Hospital of Zhengzhou Zhengzhou 450003 China

## Abstract

Correction for ‘MiR-132 enhances proliferation and migration of HaCaT cells by targeting TIMP3’ by Lina Jiang *et al.*, *RSC Adv.*, 2019, **9**, 21125–21133, DOI: 10.1039/C8RA10552A.

The authors regret that the name of one of the authors (Xiaomei Zhai) was shown incorrectly in the original article. The corrected author list is as shown above.

The Royal Society of Chemistry apologises for these errors and any consequent inconvenience to authors and readers.

## Supplementary Material

